# APLG-Net: an anatomy-guided local-global hybrid network with progression-aware supervision for structural MRI-based NC/MCI/AD classification

**DOI:** 10.3389/fneur.2026.1885491

**Published:** 2026-07-15

**Authors:** Bin Shi, Zhimin Wang, Jing Lian, Zhaorui Yang, Xiaona Zuo

**Affiliations:** 1 School of Information Science and Engineering, Lanzhou University, Lanzhou, Gansu, China; 2 Gansu Provincial Hospital, Lanzhou, Gansu, China; 3 School of Electronic and Information Engineering, Lanzhou Jiaotong University, Lanzhou, Gansu, China

**Keywords:** ADNI, Alzheimer's disease, cross-attention, local-global learning, mild cognitive impairment, ordinal regression, progression-aware supervision, structural MRI

## Abstract

**Introduction:**

Structural MRI-based Alzheimer's disease classification remains challenging due to subtle anatomical variations and the intermediate nature of mild cognitive impairment (MCI).

**Methods:**

We propose APLG-Net, an anatomy-guided local-global hybrid network with progression-aware supervision for NC/MCI/AD classification. The model integrates a global whole-brain encoder and a local ROI-based encoder, followed by cross-attention fusion and vector-gated integration. An ordinal supervision strategy is introduced to model disease progression.

**Results:**

On the ADNI dataset, APLG-Net achieves 87.1% accuracy, 86.4% balanced accuracy, 86.8% Macro-F1, and 85.6% MCI F1, outperforming CNN-based, Transformer-based, and hybrid baselines.

**Discussion:**

The results demonstrate that incorporating anatomical priors, local-global feature interaction, and ordinal supervision significantly improves MCI discrimination and overall classification robustness.

## 1 Introduction

Alzheimer's disease (AD) is one of the most common causes of dementia and is characterized by progressive cognitive decline and neurodegeneration. Mild cognitive impairment (MCI) is often considered a transitional stage between normal aging and clinically diagnosed AD, although it is heterogeneous and not all MCI subjects progress to dementia (1, 2). Accurate discrimination among normal controls (NC), MCI, and AD is therefore important for early risk assessment, patient stratification, and the development of disease-modifying interventions. Compared with binary AD/NC classification, the three-class NC/MCI/AD setting is more clinically meaningful but also more challenging because MCI lies between the two endpoints of the disease spectrum.

Structural magnetic resonance imaging (sMRI), particularly T1-weighted MRI, provides non-invasive information about brain morphology and has been widely used in AD-related computer-aided diagnosis (3, 4). AD progression is associated with progressive structural degeneration in multiple brain regions, particularly within the medial temporal lobe. Neuropathological and neuroimaging studies have consistently demonstrated early atrophy of the hippocampus, entorhinal cortex, and amygdala during the transition from normal cognition to MCI and AD dementia, accompanied by ventricular enlargement and distributed cortical alterations. (2, 5). These imaging patterns suggest that an effective model should capture both localized disease-sensitive anatomical abnormalities and broader whole-brain structural context.

Recent advances in artificial intelligence have enabled automated analysis of structural MRI for computer-aided AD diagnosis and risk assessment. Deep learning has substantially advanced MRI-based AD classification by enabling end-to-end representation learning from volumetric images (4, 6). Three-dimensional convolutional neural networks (3D CNNs) can learn hierarchical spatial features from MRI volumes, while Transformer-based and hybrid CNN-Transformer models further improve long-range contextual modeling through attention mechanisms (7–9). Recent studies have also explored attention mechanisms, self-supervised pre-training, multimodal fusion, and domain-aware brain MRI representation learning to improve generalization and reduce reliance on large labeled datasets (10–13). Despite this progress, two limitations remain important for structural MRI-based NC/MCI/AD classification.

First, many whole-brain models learn global representations from the entire MRI volume, but subtle abnormalities in small AD-related structures may be weakened by downsampling and global pooling. Conversely, region-of-interest (ROI)-based models focus on disease-sensitive anatomical regions, but they may lose whole-brain context and ignore distributed structural changes. Since AD-related neurodegeneration is both regional and network-level, local and global information should be modeled in a complementary manner rather than treated as mutually exclusive alternatives.

Second, many existing MRI-based classification frameworks model NC, MCI, and AD as independent diagnostic categories and optimize a standard multi-class cross-entropy objective. This formulation is useful for discriminative classification but does not explicitly encode the natural disease-stage ordering NC < MCI < AD. As a result, the learned representation may separate class labels without preserving the clinical progression relationship. This issue is particularly relevant for MCI, which is the most ambiguous category and often shares imaging characteristics with both NC and AD.

To address these issues, we propose APLG-Net, an anatomy-guided local-global hybrid network with progression-aware supervision for structural MRI-based NC/MCI/AD classification. APLG-Net contains a global branch for whole-brain representation learning and a local branch for ROI-based feature extraction from AD-related anatomical regions. The local-global fusion module uses cross-attention to integrate disease-sensitive ROI information with whole-brain structural context, followed by vector-gated adaptive fusion to balance local anatomical features and global brain representations. In addition, a progression-aware ordinal supervision objective is introduced to encode the ordered relationship among NC, MCI, and AD. This auxiliary supervision encourages the fused representation to reflect the disease continuum and improves the modeling of intermediate-stage subjects.

The main contributions of this work are summarized as follows:

- We propose an anatomy-guided local-global hybrid framework for structural MRI-based NC/MCI/AD classification, combining whole-brain context and AD-sensitive ROI features in a unified model.
- We design a local-global fusion strategy that integrates disease-sensitive ROI information with whole-brain structural context through cross-attention and adaptive feature fusion.
- We introduce progression-aware ordinal supervision to explicitly encode the disease-stage order among NC, MCI, and AD without requiring additional clinical scores.
- We conduct experiments on the ADNI cohort and demonstrate that APLG-Net outperforms representative CNN-, Transformer-, hybrid, attention-based, and pre-training-based baselines, with particularly clear gains in MCI discrimination.

## 2 Related work

### 2.1 Structural MRI-based Alzheimer's disease classification

Alzheimer's disease is a progressive neurodegenerative disorder characterized by cognitive decline and structural brain alterations. Mild cognitive impairment (MCI) is commonly regarded as an intermediate clinical stage between normal aging and AD, and its accurate identification is important for early risk assessment and disease management (1, 2). Structural magnetic resonance imaging (sMRI), especially T1-weighted MRI, provides non-invasive measurements of brain morphology and has therefore been widely used for computer-aided NC/MCI/AD classification (3).

Traditional machine learning studies often relied on handcrafted morphometric features, such as cortical thickness, regional volume, gray matter density, or shape descriptors extracted from predefined anatomical regions. These methods are interpretable and can be directly linked to known AD-related structures, including the hippocampus, entorhinal cortex, amygdala, medial temporal lobe, and ventricular regions. However, handcrafted representations are usually limited by feature engineering and may not fully capture high-dimensional spatial patterns distributed across the whole brain.

Deep learning methods have reduced the need for manual feature design by learning discriminative representations directly from MRI volumes (4, 6). Three-dimensional convolutional neural networks (3D CNNs) have been widely adopted because they can model local spatial patterns in volumetric data while preserving the anatomical structure of the input image. CNN-based models are effective in extracting hierarchical local features, but their receptive fields and convolutional inductive bias may limit their ability to explicitly model long-range dependencies among distant brain regions. This limitation is relevant for AD classification, where both regional atrophy and distributed whole-brain structural changes may contribute to diagnosis.

### 2.2 Whole-brain and ROI-based deep learning

Existing MRI-based deep learning methods can be broadly divided into whole-brain approaches and ROI-based approaches. Whole-brain methods use the complete MRI volume as input and aim to learn global structural representations. These methods preserve spatial context across cortical and subcortical regions and can capture large-scale patterns such as global atrophy and ventricular enlargement. Nevertheless, because whole-brain inputs are high dimensional, aggressive downsampling is often required for computational feasibility. Subtle abnormalities in small disease-sensitive structures may consequently be weakened during representation learning.

ROI-based methods focus on selected anatomical regions known to be associated with AD progression. By cropping or segmenting local patches around disease-sensitive structures, these methods can retain fine-grained local morphology and reduce irrelevant background information. In particular, medial temporal lobe structures such as the hippocampus and entorhinal cortex are closely related to memory impairment and AD pathology (2, 5). However, ROI-only methods may ignore broader whole-brain context. A local abnormality can have different diagnostic implications depending on the subject's global anatomical pattern, age-related changes, or distributed atrophy profile.

These observations suggest that whole-brain and ROI-based representations are complementary. A robust model should preserve global anatomical context while explicitly emphasizing local disease-sensitive regions. This motivates local-global hybrid architectures that jointly learn whole-brain and regional representations. In this work, APLG-Net follows this direction by combining a global branch for whole-brain representation learning and an anatomy-guided local branch for ROI encoding.

### 2.3 Attention and hybrid network architectures

Transformer and attention-based architectures have recently attracted increasing interest in medical image analysis. Compared with convolutional operations, self-attention can model pairwise relationships among tokens and is therefore useful for capturing long-range dependencies (7–9). In 3D medical imaging, however, directly applying a Transformer to dense volumetric tokens is computationally expensive. Hybrid CNN-Transformer models provide a practical compromise: CNN layers first extract low-level features and reduce spatial resolution, and Transformer blocks then model contextual interactions among compact tokens.

For AD classification, attention mechanisms are particularly useful because diagnostic evidence may be distributed across multiple brain regions. Self-attention can describe relationships among whole-brain tokens, whereas cross-attention can explicitly connect local ROI features with global contextual information. Simple fusion operations such as concatenation or summation may combine feature vectors, but they do not explicitly determine which global regions are relevant to each ROI or how much local and global information should contribute to the final representation. Therefore, an adaptive fusion mechanism is needed to integrate regional evidence and whole-brain context more effectively.

APLG-Net addresses this issue with a local-global fusion module. The module first uses cross-attention to enhance disease-sensitive ROI features using whole-brain structural context, allowing local anatomical patterns to be interpreted together with global brain organization. It then employs a vector-gated fusion mechanism to adaptively balance disease-sensitive local information and whole-brain structural context for each subject. This design aims to go beyond static feature concatenation and to support subject-specific integration of complementary diagnostic cues.

### 2.4 Progression-aware learning for ordered disease stages

Most deep learning methods formulate NC/MCI/AD diagnosis as a standard multi-class classification problem. Under this setting, cross-entropy supervision encourages the model to separate the three categories, but it does not explicitly encode their natural clinical order. In other words, the model is trained to recognize that NC, MCI, and AD are different categories, but it is not directly informed that MCI lies between NC and AD along a disease progression continuum.

This limitation is especially important for MCI classification. MCI subjects are heterogeneous and often share imaging characteristics with both normal controls and AD patients. Treating the three diagnostic stages as unrelated classes may lead to unstable intermediate-stage representations. Ordinal learning provides a way to introduce stage ordering into the training objective (14, 15). By encoding the relationship NC < MCI < AD, the model can be encouraged to organize learned features along a clinically meaningful progression direction.

In this study, we introduce progression-aware supervision as an auxiliary ordinal task. The original diagnostic labels are converted into binary ordinal labels, with NC encoded as [0, 0], MCI as [1, 0], and AD as [1, 1]. This supervision does not require additional clinical scores, but it provides an explicit constraint on disease-stage ordering. Combined with standard classification supervision, it encourages the fused representation to preserve both categorical separability and progression consistency.

## 3 Materials and methods

### 3.1 Overview of APLG-Net

**Figure 1:**
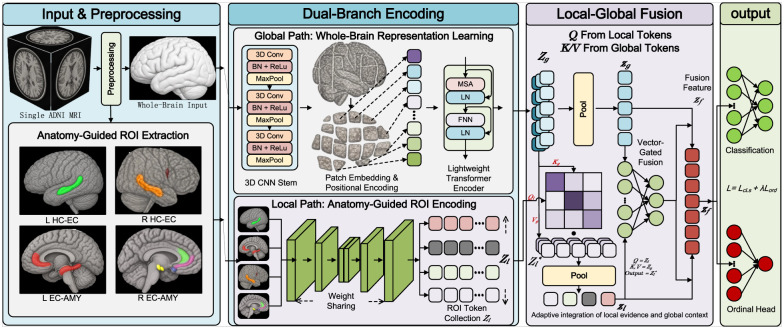
Overview of the proposed APLG-Net framework. The model first constructs a whole-brain input and anatomy-guided ROI patches from a single structural MRI scan. The global branch learns whole-brain structural representations, whereas the local branch encodes disease-sensitive ROI features with shared weights. The local-global fusion module enhances disease-sensitive ROI features using whole-brain structural context through cross-attention and adaptively combines local and global representations through vector-gated fusion. The fused feature representation is jointly used for disease classification and progression-aware ordinal supervision. Output heads and corresponding training objectives are illustrated for completeness.

We propose APLG-Net, an anatomy-guided local-global hybrid network with progression-aware supervision for structural MRI-based NC/MCI/AD classification. The feature extraction and local-global fusion architecture is shown in Figure 1. Given a single T1-weighted structural MRI scan *X*, the model constructs a preprocessed whole-brain input *X*_*g*_ and a set of anatomy-guided ROI patches $R=\left\{R_{1},\ldots ,R_{K}\right\}$. A global branch learns whole-brain structural tokens, while a local branch extracts regional features from AD-related anatomical ROIs. The local and global representations are then integrated by a fusion module consisting of cross-attention and vector-gated fusion, producing the final fused representation *z*_*f*_. The classification and ordinal supervision heads are applied to *z*_*f*_ during training, but are described separately in the training objective subsection for clarity. The overall framework is formulated in Equations 1–28.

The architecture shown in Figure 1 can be summarized as


$$\begin{array}{l} X\to \left\{X_{g},R\right\}\to \left\{Z_{g},Z_{l}\right\}\to z_{f}, \end{array}$$ (1)

where *X*_*g*_ denotes the whole-brain input, R denotes the ROI patch set, *Z*_*g*_ and *Z*_*l*_ denote the global whole-brain representation and the local ROI-based representation, respectively, and *z*_*f*_ represents the fused feature integrating both local anatomical and global contextual information. After feature fusion, *z*_*f*_ is fed into a classification head for NC/MCI/AD prediction and an auxiliary ordinal head for progression-aware supervision.

### 3.2 Input construction and anatomy-guided ROI extraction

Each input sample is a single T1-weighted structural MRI volume,


$$\begin{array}{l} X\in {\mathbb{R}}^{1\times H\times W\times D}, \end{array}$$ (2)

where *H*, *W*, and *D* denote the spatial dimensions. The task is formulated as subject-level NC/MCI/AD classification. For each subject, only one scan is retained, preferably the baseline scan, to avoid subject-level information leakage across training, validation, and test sets.

The raw MRI scans are preprocessed to improve spatial and intensity comparability across subjects. The preprocessing pipeline includes bias field correction, skull stripping, spatial registration to a standard template space, intensity normalization, and resampling to a fixed input size. The resulting whole-brain input is denoted as


$$\begin{array}{l} X_{g}=P\left(X\right), \end{array}$$ (3)

where P(·) represents the preprocessing operation. In this study, the whole-brain input size is set to 1 × 128 × 128 × 128.

To introduce anatomical prior knowledge, we extract ROI patches from AD-related structures in the standardized space. Four ROI patches are used in the default setting: left hippocampal-entorhinal ROI, right hippocampal-entorhinal ROI, left entorhinal-amygdala ROI, and right entorhinal-amygdala ROI. These regions were selected because medial temporal lobe structures are strongly associated with AD-related neurodegeneration and cognitive impairment.

These medial temporal regions were selected because they are among the earliest and most consistently affected structures in AD-related neurodegeneration. Previous neuropathological and neuroimaging studies have shown that hippocampal atrophy, entorhinal cortex degeneration, and amygdala involvement are strongly associated with memory decline and disease progression from NC to MCI and AD. The selected ROI masks are defined in the standardized MNI space according to anatomical labels corresponding to the hippocampus, entorhinal cortex, and amygdala (16, 17). The anatomical labels were obtained from FreeSurfer-based segmentation and subsequently transformed into the standardized MNI space after spatial normalization. For each hemisphere, the hippocampal-entorhinal ROI is defined as the union of the hippocampus and entorhinal cortex labels, whereas the entorhinal-amygdala ROI is defined as the union of the entorhinal cortex and amygdala labels. Left and right hemispheres are processed separately, resulting in four ROI masks. The same ROI centers are used for all subjects after spatial normalization. All ROI masks are kept in the same standardized MNI space, resolution, and voxel grid as the preprocessed MRI before patch extraction. For the *k*-th selected anatomical region, a fixed-size 3D patch is cropped from the preprocessed MNI-space MRI according to the corresponding mask:


$$\begin{array}{l} R_{k}=\text{Crop}\left(X_{g},M_{k}\right), \end{array}$$ (4)

where *M*_*k*_ is the atlas mask of the *k*-th ROI. The resulting ROI set is


$$\begin{array}{l} R=\left\{R_{1},R_{2},\ldots ,R_{K}\right\}. \end{array}$$ (5)

For each selected ROI, the centroid of the atlas-defined mask in the standardized MNI space is used as the patch center. A fixed-size 48 × 48 × 48 patch is cropped around the centroid from the preprocessed MRI. When the crop exceeds the image boundary, zero padding is applied. All ROI patches are extracted after spatial normalization to ensure anatomical correspondence across subjects. In the main configuration, *K* = 4 and each ROI patch has a size of 1 × 48 × 48 × 48.

### 3.3 Dual-branch encoding

#### 3.3.1 Global branch for whole-brain representation learning

The global branch is designed to learn whole-brain structural context from *X*_*g*_. Directly tokenizing a full 3D MRI volume for Transformer encoding is computationally expensive. Therefore, APLG-Net first uses a 3D CNN stem to extract low-level structural features and reduce spatial resolution:


$$\begin{array}{l} F_{g}=E_{g}^{cnn}\left(X_{g}\right), \end{array}$$ (6)

where $E_{g}^{cnn}\left(\cdot \right)$ denotes the global CNN stem. The stem consists of three convolutional blocks with output channels 32, 64, and 128, interleaved with normalization, nonlinear activation, and max-pooling operations. The resulting feature map is then converted into a compact sequence of global tokens:


$$\begin{array}{l} T_{g}=\text{Tokenize}\left(F_{g}\right),\;T_{g}\in {\mathbb{R}}^{N_{g}\times d}. \end{array}$$ (7)

We set the token dimension to *d* = 128 and the number of global tokens to *N*_*g*_ = 64.

To preserve spatial information, learnable positional embeddings *P*_*g*_ are added to the token sequence:


$$\begin{array}{l} T_{g}^{\prime }=T_{g}+P_{g}. \end{array}$$ (8)

The tokens are then fed into a lightweight Transformer encoder:


$$\begin{array}{l} Z_{g}=E_{g}^{trans}\left(T_{g}^{\prime }\right), \end{array}$$ (9)

where $E_{g}^{trans}\left(\cdot \right)$ contains two Transformer layers with four attention heads. The global summary feature is obtained by token pooling:


$$\begin{array}{l} z_{g}=\text{Pool}\left(Z_{g}\right),\;z_{g}\in {\mathbb{R}}^{d}. \end{array}$$ (10)

This branch captures whole-brain structural patterns, including distributed atrophy, ventricular enlargement, and contextual relationships among distant brain regions.

#### 3.3.2 Local branch for anatomy-guided ROI encoding

The local branch focuses on disease-sensitive anatomical regions. Each ROI patch *R*_*k*_ is processed by a shared 3D CNN encoder:


$$\begin{array}{l} f_{k}=E_{l}\left(R_{k}\right),\;k=1,\ldots ,K, \end{array}$$ (11)

where *E*_*l*_(·) denotes the local ROI encoder. The same encoder parameters are shared across all ROI patches to reduce model complexity and ensure that features from different ROIs are embedded into a common representation space.

The local encoder contains three 3D convolutional blocks with output channels 32, 64, and 128, followed by global average pooling and a linear projection. Each ROI is represented by a *d*-dimensional feature vector:


$$\begin{array}{l} f_{k}\in {\mathbb{R}}^{d}. \end{array}$$ (12)

The ROI features are concatenated to form the local feature set:


$$\begin{array}{l} Z_{l}=\left[f_{1};f_{2};\ldots ;f_{K}\right]\in {\mathbb{R}}^{K\times d}. \end{array}$$ (13)

Compared with the global branch, the local branch retains more fine-grained information from small AD-sensitive structures and complements the whole-brain representation.

### 3.4 Local-global fusion

The fusion module integrates local ROI evidence with whole-brain context in two steps. First, cross-attention is used to update local ROI tokens by querying global tokens. Second, a vector gate adaptively balances the context-enhanced local feature and the global summary feature.

In the cross-attention layer, ROI-based local features are used as queries, while whole-brain features serve as keys and values:


$$\begin{array}{l} Q_{l}=Z_{l}W_{Q},\;K_{g}=Z_{g}W_{K},\;V_{g}=Z_{g}W_{V}, \end{array}$$ (14)

where *W*_*Q*_, *W*_*K*_, and *W*_*V*_ are learnable projection matrices. The attention weights are computed as


$$\begin{array}{l} A=\text{Softmax}\left(\frac{Q_{l}K_{g}^{T}}{\sqrt{d}}\right), \end{array}$$ (15)

and the context-enhanced ROI features are obtained by


$$\begin{array}{l} Z_{l}^{*}=AV_{g}. \end{array}$$ (16)

$$\begin{array}{l} Z_{l}^{*}=\text{CrossAttn}\left(Q=Z_{l},K=Z_{g},V=Z_{g}\right). \end{array}$$ (17)

In implementation, the cross-attention layer is implemented as a multi-head attention block with four attention heads, residual connection, layer normalization, and dropout. Layer normalization is applied before attention, the attention output is added back to the input ROI tokens through a residual connection, and dropout with a rate of 0.1 is used to reduce overfitting. This operation allows each ROI token to selectively aggregate relevant whole-brain contextual information.

The enhanced ROI features are aggregated into a local summary representation using mean pooling across ROI features:


$$\begin{array}{l} z_{l}=\text{Pool}\left(Z_{l}^{*}\right),\;z_{l}\in {\mathbb{R}}^{d}. \end{array}$$ (18)

To adaptively integrate local ROI information and whole-brain structural context, the two feature representations are concatenated and passed through a gating multilayer perceptron (MLP):


$$\begin{array}{l} \alpha =\sigma \left(\text{MLP}\left(\left[z_{l},z_{g}\right]\right)\right), \end{array}$$ (19)

where σ(·) denotes the sigmoid function and α∈[0, 1]^*d*^ is a vector-valued gate. The gate MLP is implemented as Linear(2*d, d*), ReLU, dropout with a rate of 0.1, Linear(*d, d*), and sigmoid activation. The final fused feature is defined as


$$\begin{array}{l} z_{f}=\alpha ⊙z_{l}+\left(1-\alpha \right)⊙z_{g}, \end{array}$$ (20)

where ⊙ denotes element-wise multiplication. The vector gate enables subject-specific and dimension-wise balancing between local anatomical evidence and global structural context.

### 3.5 Dual-task prediction and training objective

The fused feature *z*_*f*_ is used by two output heads. The classification head predicts the probabilities of NC, MCI, and AD:


$$\begin{array}{l} \hat{p}=\text{Softmax}\left(W_{c}z_{f}+b_{c}\right), \end{array}$$ (21)

where $\hat{p}=\left[p_{NC},p_{MCI},p_{AD}\right]$. The classification loss is the cross-entropy loss:


$$\begin{array}{l} L_{cls}=-\overset{3}{\underset{c=1}{\sum }}y_{c}\log{\hat{p}}_{c}. \end{array}$$ (22)

When class imbalance is considered, class weights can be incorporated into the same loss.

To encode the ordered relationship among diagnostic stages, we introduce an auxiliary progression-aware ordinal classification head inspired by ordinal regression (18, 19). The ordinal labels are automatically converted from the diagnostic labels and do not require additional clinical scores. For the three ordered stages NC < MCI < AD, the labels are encoded as follows:


$$\begin{array}{l} NC\to \left[0,0\right],\text{ MCI}\to \left[1,0\right],\text{ AD}\to \left[1,1\right]. \end{array}$$ (23)

The two ordinal outputs are interpreted as cumulative probabilities corresponding to *P*(*y*>0) and *P*(*y*>1), respectively. The ordinal head outputs two logits, which are transformed by sigmoid activation:


$$\begin{array}{l} \hat{q}=\sigma \left(W_{o}z_{f}+b_{o}\right),\;\hat{q}\in {\mathbb{R}}^{2}. \end{array}$$ (24)

The ordinal loss is defined as binary cross-entropy over the two cumulative binary targets:


$$\begin{array}{l} L_{ord}=-\overset{2}{\underset{j=1}{\sum }}\left[y_{j}^{ord}\log{\hat{q}}_{j}+\left(1-y_{j}^{ord}\right)\log\left(1-{\hat{q}}_{j}\right)\right]. \end{array}$$ (25)

Because the target encoding is cumulative, the expected outputs follow the ordered pattern [0, 0], [1, 0], and [1, 1] from NC to MCI and AD. The corresponding progression score is computed as


$$\begin{array}{l} ŝ={\hat{q}}_{1}+{\hat{q}}_{2}, \end{array}$$ (26)

where values close to 0, 1, and 2 correspond to NC, MCI, and AD-like stages, respectively. The ordinal head is used as an auxiliary training branch and does not replace the classification head.

The final training objective combines classification supervision and progression-aware ordinal supervision:


$$\begin{array}{l} L=L_{cls}+\lambda L_{ord}, \end{array}$$ (27)

where λ controls the contribution of the ordinal loss. In the main experiments, λ is set to 0.5. This value provided a stable balance between classification optimization and ordinal supervision in preliminary experiments, and similar performance trends were observed within a moderate range of λ values. During inference, the primary diagnostic output is obtained from the classification head by


$$\begin{array}{l} ŷ=\arg\underset{c}{\max}{\hat{p}}_{c}, \end{array}$$ (28)

and the progression score ŝ is used only as an auxiliary indicator for analyzing disease-stage ordering.

## 4 Experiments

### 4.1 Dataset and pre-processing

#### 4.1.1 Dataset construction and label definition

Experiments were conducted on T1-weighted structural MRI scans from the Alzheimer's Disease Neuroimaging Initiative (ADNI) database. ADNI data were collected from ADNI-1, ADNI-GO, ADNI-2, and ADNI-3. The task was defined as a three-class subject-level classification problem, including normal control (NC), mild cognitive impairment (MCI), and Alzheimer's disease (AD). For each subject, one baseline or first-available T1-weighted structural MRI scan was retained to construct a subject-level cohort and avoid longitudinal information leakage.

Diagnostic labels were determined according to the clinical diagnosis recorded at the same visit as the selected MRI scan. For the three-class classification task, early mild cognitive impairment and late mild cognitive impairment were merged into a unified MCI category, while cognitively normal and Alzheimer's disease subjects were assigned to the NC and AD categories, respectively. When the baseline scan was unavailable or failed preprocessing, the earliest subsequent visit with both a valid T1-weighted MRI scan and a matched clinical diagnosis was used. Subjects without a valid image-label pair after this procedure were excluded.

Both 1.5T and 3T MRI acquisitions were included to preserve the heterogeneous multi-protocol characteristics of ADNI. All scans were processed using the same preprocessing pipeline. Quality control was performed after preprocessing, and scans with failed spatial normalization, incomplete brain coverage, severe motion or imaging artifacts, or visually unacceptable skull stripping were excluded before final cohort construction. All training, validation, and testing partitions were generated at the subject level, and the corresponding subject IDs were fixed and archived to ensure reproducibility and prevent subject overlap across data splits.

The final cohort contained 1,200 subjects, including 350 NC, 550 MCI, and 300 AD subjects. The dataset was divided into training, validation, and test sets using a stratified subject-level split with a ratio of 70%/10%/20%. The cohort statistics are summarized in Table 1. The MCI group was larger than the NC and AD groups, reflecting the common distribution of ADNI-based NC/MCI/AD classification studies.

**Table 1:** Demographic characteristics and subject-level train/validation/test distribution of the ADNI cohort.

Group	Total	Train	Val	Test	Age, years	Female, n (%)
NC	350	245	35	70	73.1 ± 6.3	184 (52.6%)
MCI	550	385	55	110	73.8 ± 7.1	232 (42.2%)
AD	300	210	30	60	75.2 ± 7.4	143 (47.7%)
Total	1,200	840	120	240	73.9 ± 7.0	559 (46.6%)

#### 4.1.2 Image pre-processing and ROI extraction

All MRI scans were preprocessed using the pipeline described in Section 3.2, including bias field correction, skull stripping, spatial registration to the standard template space, intensity normalization, and resampling to a fixed input size of 1 × 128 × 128 × 128. Anatomy-guided ROI patches were extracted from AD-related medial temporal lobe regions. The default setting used four ROIs: left hippocampal-entorhinal ROI, right hippocampal-entorhinal ROI, left entorhinal-amygdala ROI, and right entorhinal-amygdala ROI. Each ROI patch was cropped with a size of 1 × 48 × 48 × 48.

### 4.2 Experimental setup

#### 4.2.1 Implementation and training details

The main implementation and hyperparameter settings are listed in Table 2. APLG-Net used a token dimension of 128, 64 global tokens, two Transformer layers, and four attention heads. The global branch was initialized with a 3D CNN stem with channel dimensions 32–64–128, while the local branch used a shared 3D CNN ROI encoder. The fusion module contained one cross-attention block followed by a vector-gated fusion module.

**Table 2:** Implementation details, model configuration, and training protocol used for APLG-Net.

Category	Setting
Data and input	
Input modality	T1-weighted structural MRI
Whole-brain input size	1 × 128 × 128 × 128
ROI number	*K* = 4
ROI patch size	1 × 48 × 48 × 48
Network architecture	
Global CNN channels	32–64–128
Token dimension	*d* = 128
Global token number	*N*_*g*_ = 64
Transformer layers	2
Attention heads	4
Local ROI encoder	Shared 3D CNN
Cross-attention blocks	1
Gate MLP	256 → 128 → 128
Classification head	128 → 64 → 3
Ordinal head	128 → 64 → 2
Training protocol	
Optimizer	AdamW
Learning rate	1 × 10^−4^
Weight decay	1 × 10^−4^
Batch size	4
Epochs	100
Scheduler	Cosine annealing
Warm-up	5 epochs
Dropout	0.1 in encoder, 0.3 in heads
Loss weight	λ = 0.5
Model selection	Best validation Macro-F1
Repeated runs	5 random seeds

The model was trained using AdamW with a learning rate of 1 × 10^−4^ and a weight decay of 1 × 10^−4^. The batch size was set to 4, and training was performed for up to 100 epochs with 5 warm-up epochs and cosine annealing. Dropout was set to 0.1 in the encoder and 0.3 in the classification heads. The loss weight for the ordinal supervision was set to λ = 0.5. The best model was selected according to validation Macro-F1.

#### 4.2.2 Evaluation metrics and baseline methods

We evaluated all methods using Accuracy, Balanced Accuracy, Macro-F1, Macro-AUC, and class-wise precision, recall, and F1 score. Macro-F1 and Balanced Accuracy were used as the primary evaluation metrics because they are less sensitive to class imbalance than overall Accuracy. Macro-AUC was computed using the one-vs-rest macro-average strategy. Because MCI is the intermediate and most challenging stage in NC/MCI/AD classification, MCI F1 and MCI recall were treated as key indicators. All results are reported as mean ± standard deviation over five random seeds.

APLG-Net was compared with representative CNN-, Transformer-, hybrid-network, attention-based, and pre-training-based baselines, including 3D CNN, 3D ResNet-18 (20, 21), 3D DenseNet (22), 3D ViT (8), 3D SwinT (9), CNN-Transformer, MedicalNet-ResNet (23, 24), Att-3D DenseNet (11), SSL-3D Swin (25, 26), Domain-aware 3D Swin (12), and 3D-CNN-VSwinFormer (27). Ablation studies were further conducted to evaluate the contribution of the global branch, local branch, cross-attention, vector-gated fusion, and progression-aware ordinal supervision.

For a fair comparison, all baseline methods used the same subject-level train/validation/test split, the same MRI preprocessing pipeline, the same whole-brain input size of 1 × 128 × 128 × 128, the same evaluation metrics, and the same five repeated random seeds. The best checkpoint for each method was selected according to validation Macro-F1. For pre-trained baselines, the full network was fine-tuned on the ADNI training set without freezing any layers. The pre-trained weights were obtained from public medical-image or brain-MRI pre-training sources. To ensure a fair comparison, the same preprocessing pipeline, optimizer settings, learning-rate schedule, batch size, and early stopping strategy were applied consistently across all competing methods. No test-set images or labels were used during pre-training, hyperparameter tuning, or model selection.

Statistical comparisons were performed over the repeated runs. For model-level metrics, paired two-sided Wilcoxon signed-rank tests were used to compare APLG-Net with the strongest baseline across the five random seeds. For progression scores, group differences among NC, MCI, and AD were assessed using the Kruskal–Wallis test followed by post-hoc pairwise comparisons with Bonferroni correction.

### 4.3 Main comparison results

#### 4.3.1 Overall comparison with competing methods

Table 3 presents the main comparison results. APLG-Net achieved the best overall performance among all compared methods, with an Accuracy of 87.1 ± 0.7%, a Balanced Accuracy of 86.4 ± 0.8%, a Macro-F1 of 86.8 ± 0.7%, and a Macro-AUC of 0.938 ± 0.005. Compared with the strongest baseline, 3D-CNN-VSwinFormer, APLG-Net improved Accuracy by 1.1 percentage points, Balanced Accuracy by 1.4 percentage points, Macro-F1 by 1.9 percentage points, and MCI F1 by 3.0 percentage points.

**Table 3:** Performance comparison with representative CNN, Transformer, hybrid, attention-based, and pre-training-based baselines on the ADNI NC/MCI/AD classification task.

Method	Type	Accuracy (%)	Balanced accuracy (%)	Macro-F1 (%)	Macro-AUC	MCI F1 (%)
3D CNN	Basic CNN	78.4 ± 1.5	76.9 ± 1.7	76.1 ± 1.6	0.865 ± 0.011	70.2 ± 2.1
3D ResNet-18	CNN	81.6 ± 1.2	80.1 ± 1.4	79.6 ± 1.3	0.889 ± 0.009	75.8 ± 1.8
3D DenseNet	CNN	82.0 ± 1.1	80.5 ± 1.3	80.2 ± 1.2	0.896 ± 0.008	76.3 ± 1.6
3D ViT	Transformer	80.8 ± 1.4	79.4 ± 1.5	78.9 ± 1.5	0.887 ± 0.010	74.5 ± 1.9
3D SwinT	Transformer	83.1 ± 1.0	81.9 ± 1.2	81.5 ± 1.1	0.910 ± 0.007	78.4 ± 1.5
CNN-Transformer	Hybrid	84.0 ± 0.9	82.8 ± 1.1	82.6 ± 1.0	0.917 ± 0.007	79.6 ± 1.4
MedicalNet-ResNet	Pre-trained CNN	84.5 ± 0.9	83.3 ± 1.0	83.1 ± 0.9	0.920 ± 0.007	80.3 ± 1.4
Att-3D DenseNet	Attention CNN	84.8 ± 0.9	83.7 ± 1.0	83.5 ± 0.9	0.922 ± 0.006	80.7 ± 1.3
SSL-3D Swin	SSL transformer	85.2 ± 0.8	84.1 ± 0.9	83.9 ± 0.9	0.925 ± 0.006	81.3 ± 1.2
Domain-aware 3D Swin	Brain-MRI pre-training	85.7 ± 0.8	84.8 ± 0.9	84.6 ± 0.8	0.929 ± 0.006	82.2 ± 1.2
3D-CNN- VSwinFormer	Recent hybrid	86.0 ± 0.8	85.0 ± 0.8	84.9 ± 0.8	0.930 ± 0.006	82.6 ± 1.1
APLG-Net	Ours	**87.1** **±0.7**	**86.4** **±0.8**	**86.8** **±0.7**	**0.938** **±0.005**	**85.6** **±1.0**

Bold values indicate the best performance among all compared methods for each metric.

The ranking of the compared methods shows several consistent trends. First, the simple 3D CNN obtained the lowest performance, indicating that shallow convolutional modeling is insufficient for capturing the complex structural patterns required for NC/MCI/AD discrimination. Second, stronger CNN backbones such as 3D ResNet-18 and 3D DenseNet improved the results, but their MCI F1 scores remained lower than 77%. Third, Transformer and hybrid models generally outperformed pure CNN models, suggesting that contextual modeling is beneficial for structural MRI classification. Fourth, pre-training-based and attention-enhanced baselines further improved performance, with DDomain-aware 3D Swin and 3D-CNN-VSwinFormer achieving the strongest baseline results. Nevertheless, APLG-Net still achieved the best performance, suggesting that explicit anatomy-guided local-global fusion and disease-order supervision provide complementary gains beyond stronger backbone design or pre-training alone.

**Figure 2:**
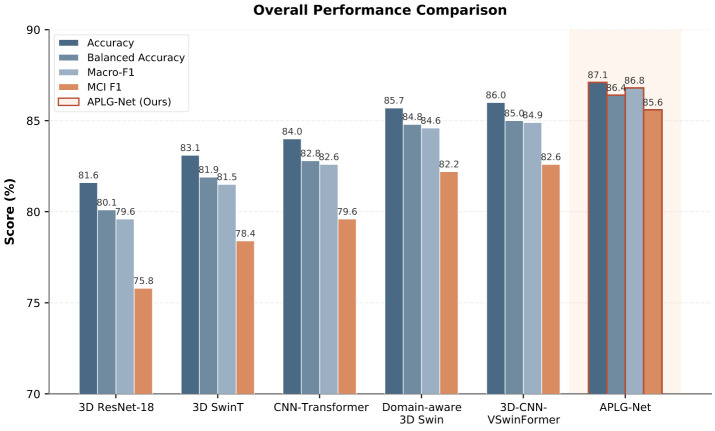
Overall performance comparison between APLG-Net and representative baseline methods. The figure shows accuracy, balanced accuracy, Macro-F1, and MCI F1 for each method.

The overall performance comparison is visualized in Figure 2. The figure provides a more direct comparison across the major metrics and shows that the improvement of APLG-Net is not limited to a single score. In particular, the gain in MCI F1 is larger than the gain in overall Accuracy, which is important because high overall accuracy can sometimes be dominated by easier NC or AD samples. This pattern suggests that the proposed method improves the clinically more challenging intermediate-stage classification rather than merely increasing performance on the more separable classes.

#### 4.3.2 Class-wise performance analysis

Table 4 reports class-wise precision, recall, and F1 score for representative strong baselines and APLG-Net. For all compared methods, MCI was more difficult to classify than NC and AD, as reflected by lower MCI F1 scores. This is consistent with the intermediate nature of MCI and the overlap of MCI imaging patterns with both normal aging and AD.

**Table 4:** Class-wise diagnostic performance of representative strong baselines and APLG-Net.

Method	NC Prec.	NC Rec.	NC F1	MCI Prec.	MCI Rec.	MCI F1	AD Prec.	AD Rec.	AD F1
3D ResNet-18	80.8	82.9	81.8	76.5	75.1	75.8	82.6	82.1	82.3
CNN-Transformer	84.8	85.7	85.2	80.3	78.9	79.6	85.6	85.0	85.3
Domain-aware 3D Swin	86.1	86.8	86.4	82.8	81.7	82.2	86.5	86.1	86.3
3D-CNN- VSwinFormer	86.5	87.1	86.8	83.3	81.9	82.6	86.9	86.5	86.7
APLG-Net	**88.2**	**88.9**	**88.5**	**86.1**	**85.1**	**85.6**	**88.0**	**88.5**	**88.2**

Bold values indicate the best performance among all compared methods for each metric.

APLG-Net achieved the best class-wise performance across all three diagnostic groups. In particular, the MCI precision, recall, and F1 reached 86.1%, 85.1%, and 85.6%, respectively. Compared with 3D-CNN-VSwinFormer, APLG-Net improved MCI precision by 2.8 percentage points, MCI recall by 3.2 percentage points, and MCI F1 by 3.0 percentage points. These results indicate that the proposed model is especially effective in improving intermediate-stage discrimination.

The class-wise results also show that APLG-Net improves NC and AD performance while preserving a larger relative advantage for MCI. NC and AD are usually easier to separate because they correspond to the two ends of the disease spectrum, whereas MCI is a heterogeneous group with mixed normal-like and AD-like imaging characteristics. The simultaneous improvement in MCI precision and recall indicates that APLG-Net reduces both over-diagnosis and under-diagnosis of MCI. This supports the design motivation of combining local anatomical evidence with whole-brain context and ordinal supervision.

#### 4.3.3 Confusion matrix analysis

**Figure 3:**
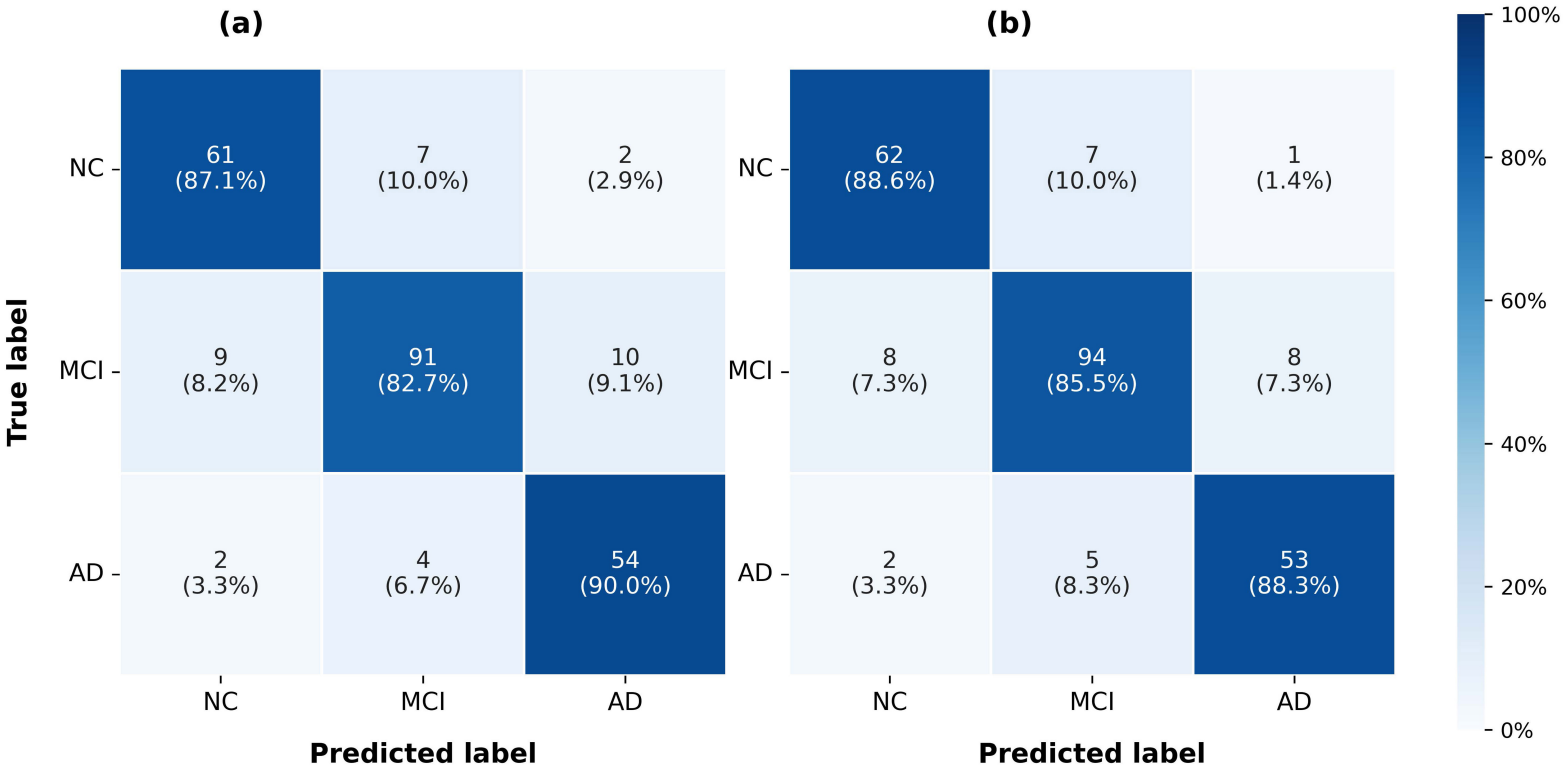
Confusion matrix comparison between 3D-CNN-VSwinFormer and APLG-Net. APLG-Net reduced the misclassification of MCI subjects and improved class balance compared with 3D-CNN-VSwinFormer, indicating improved discrimination of the intermediate disease stage. The confusion matrices are shown for the run whose test Macro-F1 was closest to the average performance across the five random seeds, while Table 4 reports mean class-wise performance over all runs. **(a)** 3D-CNN-VSwinFormer. **(b)** APLG-Net.

Figure 3 shows the confusion matrix comparison between the strongest baseline and APLG-Net. The main improvement of APLG-Net was observed in the MCI group, where fewer samples were confused with NC or AD. This supports the hypothesis that combining local ROI evidence, whole-brain context, and ordinal disease-stage supervision helps stabilize the representation of intermediate-stage subjects.

The confusion pattern is also consistent with the class-wise metrics in Table 4. Misclassification between NC and MCI reflects the difficulty of detecting subtle structural changes at an early stage, while misclassification between MCI and AD reflects the overlap between progressive MCI and clinically diagnosed AD. By reducing errors on both sides of the MCI class, APLG-Net appears to learn a more ordered representation of the disease continuum. This behavior is desirable for MRI-based AD classification because the intermediate stage is often the most clinically informative but also the most ambiguous.

### 4.4 Ablation studies

Table 5 summarizes the ablation results. The global branch only model outperformed the local branch only model, indicating that whole-brain structural context provides a strong diagnostic foundation. However, the full APLG-Net clearly outperformed both single-branch variants, demonstrating the complementarity between global context and anatomy-guided local details.

**Table 5:** Ablation analysis of the architectural components and progression-aware supervision in APLG-Net.

Variant	Accuracy (%)	Balanced accuracy (%)	Macro-F1 (%)	Macro-AUC	MCI F1 (%)
Global branch only	83.2 ± 1.0	81.8 ± 1.2	81.4 ± 1.1	0.908 ± 0.008	78.1 ± 1.6
Local branch only	80.9 ± 1.3	79.2 ± 1.4	78.8 ± 1.4	0.886 ± 0.010	75.4 ± 1.9
Simple concatenation fusion	84.3 ± 0.9	83.0 ± 1.0	82.8 ± 1.0	0.916 ± 0.007	79.7 ± 1.5
w/o Cross-attention	84.9 ± 0.9	83.6 ± 1.0	83.3 ± 0.9	0.921 ± 0.007	80.8 ± 1.4
w/o Vector gate	85.2 ± 0.8	84.0 ± 0.9	83.8 ± 0.9	0.924 ± 0.006	81.2 ± 1.3
w/o Ordinal supervision	85.5 ± 0.8	84.4 ± 0.9	84.2 ± 0.8	0.927 ± 0.006	81.6 ± 1.2
Full APLG-Net	**87.1** **±0.7**	**86.4** **±0.8**	**86.8** **±0.7**	**0.938** **±0.005**	**85.6** **±1.0**

Bold values indicate the best performance among all compared methods for each metric.

Compared with simple concatenation fusion, the full fusion module achieved better performance, confirming that explicit local-global interaction is more effective than direct feature joining. Removing cross-attention or vector-gated fusion reduced all major metrics, suggesting that both components contribute to the final representation. Removing ordinal supervision also degraded performance, with MCI F1 decreasing from 85.6 ± 1.0% to 81.6 ± 1.2%. This 4.0 percentage point reduction indicates that progression-aware supervision plays an important role in improving MCI discrimination.

The ablation results verify the three main design choices of APLG-Net. The comparison between global-only and local-only variants shows that global structural context is the dominant information source, but the gap between the full model and the global-only model demonstrates that ROI-level anatomical cues still provide important complementary evidence. The comparison among simple concatenation, removing cross-attention, removing vector gate, and the full fusion module further shows that local-global integration requires both interaction and adaptive weighting. Cross-attention enables ROI features to incorporate relevant whole-brain contextual information, while the vector gate adaptively balances the contribution of local and global information across feature representations.

The ordinal supervision ablation is particularly important for the central claim of this work. Although removing ordinal supervision still preserves the local-global architecture, its MCI F1 drops markedly. This suggests that architectural fusion alone is not sufficient for fully resolving the ambiguity of MCI; the additional disease-order constraint helps shape the fused representation so that MCI is positioned between NC and AD. Therefore, progression-aware supervision contributes not only to the auxiliary progression score, but also to the primary classification objective.

### 4.5 Progression score analysis

Because APLG-Net incorporates an ordinal classification head, we further analyze the learned progression score $ŝ={\hat{q}}_{1}+{\hat{q}}_{2}$. The progression score is not used to replace the classification output during inference, but instead provides an auxiliary perspective on how the model organizes subjects along the NC–MCI–AD continuum. If the model successfully learns the progression-aware objective, the learned scores are expected to increase monotonically from NC to MCI and AD.

**Figure 4:**
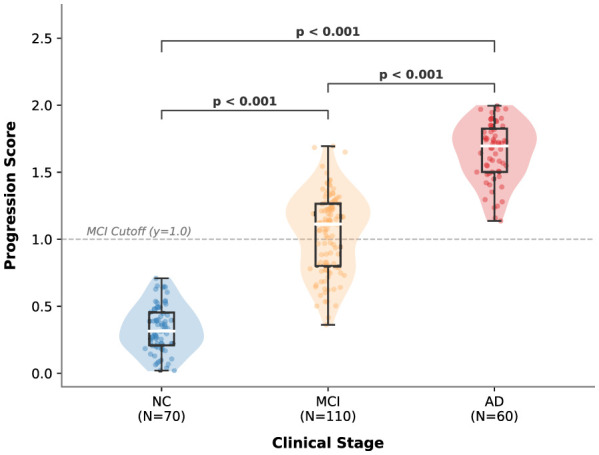
Progression score distribution estimated by the ordinal head. The learned scores follow the expected NC-MCI-AD order, consistent with disease progression. This further supports the interpretability of the progression-aware supervision. Statistical significance was assessed using Kruskal–Wallis tests with Bonferroni-corrected post-hoc comparisons.

As shown in Figure 4, the score distribution followed the expected disease-stage order, with NC showing the lowest scores, MCI occupying the intermediate range, and AD showing the highest scores. This result indicates that the ordinal head learned a meaningful progression-related representation rather than only providing an auxiliary training signal. The separation between NC and AD is clear, while the MCI distribution lies between the two groups and partially overlaps with both sides, which is consistent with the transitional nature of MCI. These scores are intended only for auxiliary supervision and interpretability analysis of disease progression patterns, rather than as quantitative clinical staging measures.

The interpretability of APLG-Net is supported by its anatomy-guided design and progression-aware representation learning. By explicitly incorporating disease-sensitive medial temporal ROIs together with whole-brain structural context, the model captures imaging patterns consistent with known AD-related neurodegeneration. The ordinal progression scores further exhibit a biologically plausible NC-MCI-AD ordering, suggesting that the learned representation reflects disease-stage continuity rather than purely categorical separation.

The intermediate distribution of MCI scores is particularly important. It suggests that APLG-Net does not simply force MCI into an isolated category, but instead represents it as a transitional stage between NC and AD. This behavior is consistent with the known clinical heterogeneity of MCI and its role as an intermediate state in Alzheimer's disease progression, and helps explain the improved MCI F1 and recall observed in the main comparison and ablation studies.

## 5 Discussion

### 5.1 Main findings

This study proposed APLG-Net, an anatomy-guided local-global hybrid network with progression-aware supervision for structural MRI-based NC/MCI/AD classification. Under the controlled experimental setting, APLG-Net achieved the best performance among the compared CNN-, Transformer-, hybrid-, attention-, and pre-training-based baselines. The improvement was most evident for MCI discrimination, which is clinically important and technically challenging because MCI lies between normal aging and AD.

The main comparison results show clear gains over representative baselines, with an Accuracy of 87.1 ± 0.7%, a Balanced Accuracy of 86.4 ± 0.8%, a Macro-F1 of 86.8 ± 0.7%, a Macro-AUC of 0.938 ± 0.005, and an MCI F1 score of 85.6 ± 1.0%. These results suggest that the proposed combination of anatomical guidance, local-global feature interaction, and progression-aware supervision is useful for structural MRI-based three-stage AD classification.

### 5.2 Effectiveness of local-global modeling

The ablation study confirms the complementarity of whole-brain and ROI-level information. The global branch is the main backbone of the model and provides whole-brain context for classification. The local branch supplies AD-sensitive regional evidence from medial temporal structures such as the hippocampus, entorhinal cortex, and amygdala. This combination is consistent with the known neuropathological progression of AD, in which local medial temporal lobe changes and broader cortical or subcortical alterations both contribute to disease characterization (2, 5).

The fusion ablations show that simple feature concatenation is not sufficient to fully exploit local-global complementarity. Cross-attention enables interaction between local ROI features and whole-brain structural context by aggregating complementary information across regions. The vector gate then adaptively balances the context-enhanced local representation and the whole-brain structural representation for each subject. This design is more flexible than static fusion strategies and is suitable for heterogeneous subjects whose diagnostic evidence may depend on different anatomical patterns.

### 5.3 Contribution of progression-aware supervision

The progression-aware ordinal objective is another important component of APLG-Net. It does not require additional clinical labels; the ordinal targets are automatically converted from the original diagnostic labels. Standard cross-entropy supervision treats NC, MCI, and AD as separate categories, whereas the ordinal objective provides disease-order guidance by encoding NC, MCI, and AD as [0, 0], [1, 0], and [1, 1].

The ablation results show that removing ordinal supervision decreased MCI F1 from 85.6 ± 1.0% to 81.6 ± 1.2%. This indicates that the ordinal objective mainly helps the representation of intermediate-stage subjects. The progression score analysis further supports this interpretation, as the learned scores increased from NC to MCI and AD. Therefore, ordinal supervision acts as a disease-order regularizer rather than a replacement for the diagnostic classification head.

### 5.4 Comparison with recent research trends

Recent AD classification studies have increasingly moved beyond conventional 3D CNNs toward attention-based networks, hybrid CNN-Transformer architectures, self-supervised or domain-aware pre-training, and multimodal fusion (10–13, 25, 28–30). In this context, APLG-Net is aligned with recent methodological trends because it uses attention for local-global interaction and introduces a disease-progression constraint beyond standard classification labels. The proposed method differs from many generic Transformer-based models by explicitly incorporating anatomical ROI guidance and by using ordinal supervision to reflect the ordered NC/MCI/AD relationship.

Nevertheless, cross-study comparisons in ADNI are difficult because reported performance depends strongly on cohort definition, diagnostic label selection, preprocessing, scan-level vs. subject-level splitting, use of longitudinal scans, modality combinations, and whether external pre-training is used. Some published studies report very high accuracy under binary AD/NC settings or slice-level evaluation, while three-class NC/MCI/AD subject-level classification is usually more difficult. Therefore, the most reliable evidence for APLG-Net is the controlled comparison under the same dataset split and preprocessing pipeline. In the revised comparison, APLG-Net was evaluated against stronger recent baselines, including pre-trained CNN, attention-enhanced CNN, self-supervised Transformer, Domain-aware 3D Swin, and 3D-CNN-VSwinFormer variants, and showed clear gains in overall and MCI-specific performance.

### 5.5 Limitations

First, the experiments were conducted on ADNI only. Although ADNI is a multi-center dataset, its acquisition protocols, scanner settings, and participant characteristics may not fully represent broader clinical populations or real-world imaging variability. External validation on independent cohorts such as OASIS or AIBL is therefore needed to further evaluate generalization across different scanners, acquisition conditions, and population distributions. Further validation on independent real-world clinical populations is required before potential clinical deployment. Second, this study used T1-weighted structural MRI only. Multimodal information such as PET, cognitive scores, genetic markers, and clinical variables may provide complementary evidence. Third, ROI extraction depends on atlas definition and registration accuracy. Fixed-size ROI patches may not fully adapt to individual anatomical variability, especially in subjects with severe atrophy or imperfect spatial normalization.

Fourth, the progression-aware supervision in this work is derived from diagnostic labels rather than longitudinal conversion outcomes or continuous clinical scores. Although the ordinal labels encode the NC–MCI–AD order without requiring additional annotations, they do not fully capture individual-level disease progression. Future work could integrate longitudinal follow-up, MCI conversion status, cognitive scales, or biomarker measurements and extend APLG-Net to multimodal and longitudinal settings.

### 5.6 Conclusion

In this work, we proposed APLG-Net for structural MRI-based NC/MCI/AD classification. The model combines whole-brain representation learning, anatomy-guided ROI encoding, cross-attention-based local-global interaction, vector-gated fusion, and progression-aware ordinal supervision. Experiments on the ADNI cohort showed that APLG-Net achieved the best performance under the controlled setting and consistently improved MCI discrimination. Ablation studies confirmed the contributions of the local branch, global branch, fusion module, and ordinal supervision. These findings suggest that combining anatomical guidance, local-global feature learning, and disease-stage ordering is a promising strategy for MRI-based Alzheimer's disease classification.

## Data Availability

The original contributions presented in the study are included in the article/supplementary material, further inquiries can be directed to the corresponding author.
